# (*R*)-3,3-Diethyl-1-(2-hy­droxy-1-phenyl­eth­yl)piperidin-2-one

**DOI:** 10.1107/S1600536812025433

**Published:** 2012-07-07

**Authors:** Oscar Romero, Johana Ramírez, Joel L. Terán, Marcos Flores-Alamo, Jorge R. Juárez

**Affiliations:** a Centro de Química, Instituto de Ciencias, Benemérita Universidad Autónoma de Puebla, 72570, Puebla, Pue., Mexico; b Facultad de Química, Universidad Nacional Autónoma de México, 04510, D.F., Mexico

## Abstract

In the title compound C_17_H_25_NO_2_, the piperidin-2-one ring adopts an envelope conformation with the C atom in the 5-position as the flap. The crystal packing is stabilized by inter­molecular O—H⋯O hydrogen bonds, building a infinite chain along the *b*-axis direction. C—H⋯π inter­actions further stabilize the crystal packing.

## Related literature

For background to the synthesis of piperidines, see: Angle & Breitenbucher (1995 ▶); Micouin *et al.* (1994 ▶); Deslongchamps *et al.* (1975) ▶. For ring conformation analysis, see: Cremer & Pople (1975 ▶).
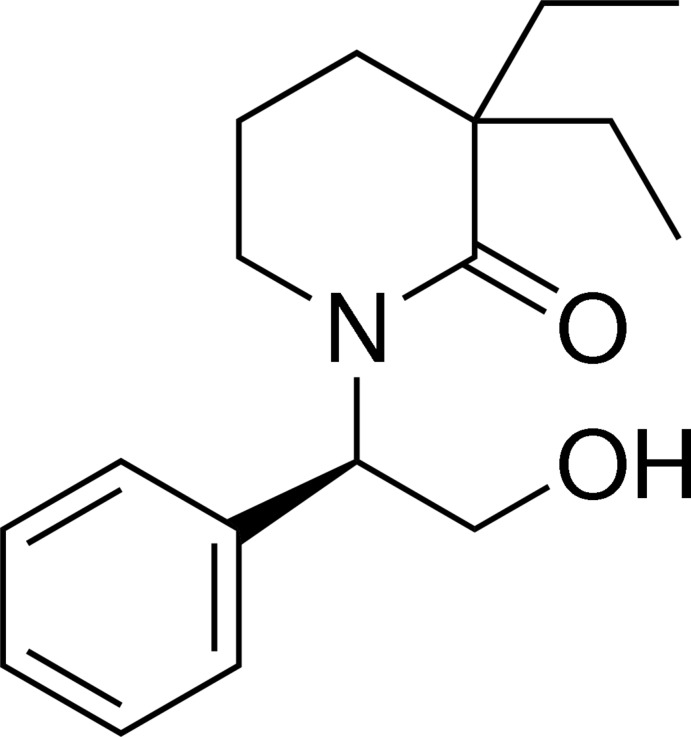


## Experimental

### 

#### Crystal data

C_17_H_25_NO_2_
*M* *_r_* = 275.38
Monoclinic, 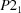

*a* = 7.5380 (3) Å
*b* = 12.6705 (6) Å
*c* = 7.9255 (4) Å
β = 91.776 (4)°
*V* = 756.61 (6) Å^3^
*Z* = 2
Mo *K*α radiation
μ = 0.08 mm^−1^
*T* = 130 K
0.35 × 0.28 × 0.13 mm

#### Data collection

Oxford Diffraction Xcalibur Atlas Gemini diffractometer
Absorption correction: analytical (*CrysAlis PRO*; Oxford Diffraction, 2009 ▶) *T*_min_ = 0.978, *T*_max_ = 0.99
5226 measured reflections
1552 independent reflections
1381 reflections with *I* > 2σ(*I*)
*R*_int_ = 0.039

#### Refinement

*R*[*F* ^2^ > 2σ(*F* ^2^)] = 0.044
*wR*(*F* ^2^) = 0.114
*S* = 1.06
1552 reflections
185 parameters
1 restraint
H atoms treated by a mixture of independent and constrained refinement
Δρ_max_ = 0.37 e Å^−3^
Δρ_min_ = −0.30 e Å^−3^

### 

Data collection: *CrysAlis CCD* (Oxford Diffraction, 2009 ▶); cell refinement: *CrysAlis CCD*; data reduction: *CrysAlis RED* (Oxford Diffraction, 2009 ▶); program(s) used to solve structure: *SHELXS97* (Sheldrick, 2008 ▶); program(s) used to refine structure: *SHELXL97* (Sheldrick, 2008 ▶); molecular graphics: *ORTEP-3 for Windows* (Farrugia, 1997 ▶); software used to prepare material for publication: *WinGX* publication routines (Farrugia, 1999 ▶).

## Supplementary Material

Crystal structure: contains datablock(s) global, I. DOI: 10.1107/S1600536812025433/bt5939sup1.cif

Structure factors: contains datablock(s) I. DOI: 10.1107/S1600536812025433/bt5939Isup2.hkl

Supplementary material file. DOI: 10.1107/S1600536812025433/bt5939Isup3.cml

Additional supplementary materials:  crystallographic information; 3D view; checkCIF report

## Figures and Tables

**Table 1:** Hydrogen-bond geometry (Å, °) *Cg*1 is the centroid of the C12–C17 ring.

*D*—H⋯*A*	*D*—H	H⋯*A*	*D*⋯*A*	*D*—H⋯*A*
O2—H1*O*⋯O1^i^	0.80 (4)	1.95 (4)	2.745 (3)	170 (4)
C4—H4*A*⋯*Cg*1^ii^	0.96	2.96	3.723 (3)	137

Symmetry codes: (i) 
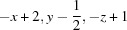
; (ii) 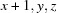.

**Fig. 1.**
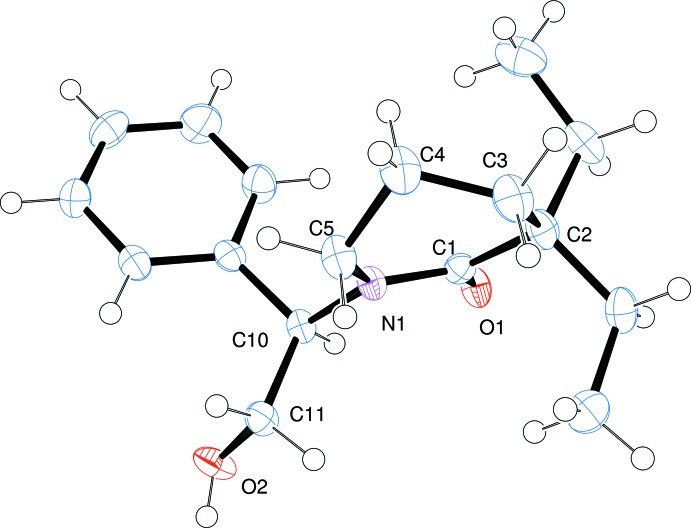
The molecular structure of title compound, with atom labels and 50% probability displacement ellipsoids for non-H atoms.

## supplementary crystallographic information

### Comment

The development of new methods for the enantioselective synthesis of piperidine derivatives by introduction of substituents at carbon positions of the heterocycle constitutes an area of curret interest (Angle & Breitenbucher, 1995). In the context of the enantioselective synthesis of 3-substituted piperidines, the enolate alkylation of the amide carbonyl of lactams derived from phenylglycinol with alkyl halides takes place with high diastereoselectivity to ultimately give enantiopure 3-alkylpiperidines in good yields. Although numerous methods have been developed for the α-alkylation, the double α-substitution in amides has been rarely studied (Micouin *et al.*, 1994; Deslongchamps *et al.*, 1975).

In the title compound C_17_H_25_NO_2_, the six membered ring N1/C1/C2/C3/C4/C5 shows an envelope conformation on C(4) with puckering parameters (Cremer & Pople, 1975) *Q* = 0.496 (3) Å, θ_2_ = 58.3 (3)°, φ_2_ = 240.7 (4)°, q_2_ = 0.422 (3) Å and q_3_ = 0.261 (3) Å. The N(1) atom in the piperidone moiety shows a planar conformation (r.m.s. deviation of N1, C1, C5 and C10 = 0.002Å). The quiral centre on C(10) shows an *R* absolute configuration with [α]_D_ = -92. Crystal packing is stabilizad by hydrogen bond interactions [O(2)—H(1O)···O(1)], building a infinite chain along *b* direction and a intermolecular C(4)—H(4 A)···π interactions making a one-dimentional chain along *a* axis. Two intramolecular interactions, C8—(H8B)···O(1) and C(10)—H10···O(1) are too observated.

### Experimental

The title compound, C_17_H_25_NO_2_, was obtained disolving (*R*)-(-)-1-(2'-hidroxy-1'-phenylethyl)piperidin-2-one (0.29 g, 1.32 mmol) in THF anhydrous and added 4.0 equiv. HMPA and 4.5 equiv. s-BuLi. The mixture was stirred for 1 h and 3 equiv. of Iodoethane was added at -78 °C, the mixture was stirred for 2.5 h. Finally, the mixture was treated with a satured solution of NH_4_Cl (4.0 ml), extracted with ethyl acetate (3x20 ml) and purified by flash chromatography (SiO_2_, AcOEt: Petroleum ether; 6:4). Yield 80%. White crystals. [*α*]_D_= -92 (*c* 1.5, CH_2_Cl_2_). IR (KBr) 1615 cm^-1^. p.f.=89–91 °C, ^1^H NMR (CDCl_3_) *δ* (p.p.m.), *J*(Hz): 0.88 (t, 3H, *J*= 7.5, 7.2 Hz), 0.96 (t, 3H, *J*=7.5, 7.2 Hz), 2.82 (m, 1H), 2.89 (m, 1H), 3.14 (m, 1H), 3.32 (br, 1*H*-OH), 4.02–4.20 (dd, 2H, *J*= 5.1, 11.1 Hz), 5.89 (dd 1H, *J*= 5.1 Hz), 7.32 (m, 5H). ^13^C NMR (CDCl_3_), 8.9, 8.9, 20.5, 28.4, 31.9, 32.2, 44.0, 46.2, 58.5, 61.8, 127.5–128.5, 137.1, 177.1.

### Refinement

All H atoms were found in a difference map. The H atom bonded to O2 was freely refined. H atoms bonded to C atoms were placed in geometrical idealized positions and refined as riding on their parent atoms, with C—H = 0.93–0.98 Å and with *U*_iso_(H) = 1.2 *U*_eq_(C) or *U*_eq_(H) = 1.5 *U*_eq_(C) for methyl groups. The absolute configuration of the chiral centre could not be determined and was set according to the starting material.

### Crystal data

**Table:**

C_17_H_25_NO_2_	*F*(000) = 300
*M**_r_* = 275.38	*D*_x_ = 1.209 Mg m^−^^3^
Monoclinic, *P*2_1_	Mo *K*α radiation, λ = 0.71073 Å
*a* = 7.5380 (3) Å	Cell parameters from 3593 reflections
*b* = 12.6705 (6) Å	θ = 3.7–26.0°
*c* = 7.9255 (4) Å	µ = 0.08 mm^−^^1^
β = 91.776 (4)°	*T* = 130 K
*V* = 756.61 (6) Å^3^	Plate, colorless
*Z* = 2	0.35 × 0.28 × 0.13 mm

### Data collection

**Table:**

Oxford Diffraction Xcalibur Atlas Gemini diffractometer	1552 independent reflections
Graphite monochromator	1381 reflections with *I* > 2σ(*I*)
Detector resolution: 10.4685 pixels mm^-1^	*R*_int_ = 0.039
ω scans	θ_max_ = 26.1°, θ_min_ = 3.7°
Absorption correction: analytical (*CrysAlis PRO*; Oxford Diffraction, 2009)	*h* = −9→9
*T*_min_ = 0.978, *T*_max_ = 0.99	*k* = −14→15
5226 measured reflections	*l* = −7→9

### Refinement

**Table:**

Refinement on *F*^2^	Primary atom site location: structure-invariant direct methods
Least-squares matrix: full	Secondary atom site location: difference Fourier map
*R*[*F*^2^ > 2σ(*F*^2^)] = 0.044	Hydrogen site location: inferred from neighbouring sites
*wR*(*F*^2^) = 0.114	H atoms treated by a mixture of independent and constrained refinement
*S* = 1.06	*w* = 1/[σ^2^(*F*_o_^2^) + (0.0844*P*)^2^] where *P* = (*F*_o_^2^ + 2*F*_c_^2^)/3
1552 reflections	(Δ/σ)_max_ < 0.001
185 parameters	Δρ_max_ = 0.37 e Å^−^^3^
1 restraint	Δρ_min_ = −0.30 e Å^−^^3^

### Special details

**Table:**

Geometry. All s.u.'s (except the s.u. in the dihedral angle between two l.s. planes) are estimated using the full covariance matrix. The cell s.u.'s are taken into account individually in the estimation of s.u.'s in distances, angles and torsion angles; correlations between s.u.'s in cell parameters are only used when they are defined by crystal symmetry. An approximate (isotropic) treatment of cell s.u.'s is used for estimating s.u.'s involving l.s. planes.
Refinement. Refinement of *F*^2^ against ALL reflections. The weighted *R*-factor *wR* and goodness of fit *S* are based on *F*^2^, conventional *R*-factors *R* are based on *F*, with *F* set to zero for negative *F*^2^. The threshold expression of *F*^2^ > 2σ(*F*^2^) is used only for calculating *R*-factors(gt) *etc*. and is not relevant to the choice of reflections for refinement. *R*-factors based on *F*^2^ are statistically about twice as large as those based on *F*, and *R*- factors based on ALL data will be even larger.

### Fractional atomic coordinates and isotropic or equivalent isotropic displacement parameters (Å^2^)

**Table:**

	*x*	*y*	*z*	*U*_iso_*/*U*_eq_	
O1	0.8432 (2)	0.61989 (14)	0.4209 (2)	0.0257 (4)	
C10	0.9135 (3)	0.4297 (2)	0.2824 (3)	0.0194 (5)	
H10	0.9956	0.4778	0.3421	0.023*	
O2	1.1120 (2)	0.29608 (16)	0.3809 (3)	0.0308 (5)	
C12	0.9617 (3)	0.4306 (2)	0.0986 (3)	0.0209 (5)	
C1	0.7126 (3)	0.56610 (19)	0.3767 (3)	0.0201 (5)	
N1	0.7335 (3)	0.47186 (16)	0.3024 (3)	0.0193 (5)	
C11	0.9290 (3)	0.3223 (2)	0.3708 (3)	0.0231 (5)	
H11A	0.8814	0.3263	0.483	0.028*	
H11B	0.8632	0.2691	0.3068	0.028*	
C13	0.9956 (4)	0.3401 (2)	0.0065 (4)	0.0293 (6)	
H13	0.9863	0.2743	0.0576	0.035*	
C8	0.5095 (4)	0.7194 (2)	0.3363 (4)	0.0325 (7)	
H8A	0.3996	0.7502	0.3735	0.039*	
H8B	0.606	0.7618	0.3835	0.039*	
C14	1.0431 (4)	0.3466 (3)	−0.1603 (3)	0.0335 (7)	
H14	1.0637	0.2852	−0.2209	0.04*	
C5	0.5857 (3)	0.4055 (2)	0.2393 (3)	0.0264 (6)	
H5A	0.5552	0.355	0.3257	0.032*	
H5B	0.6227	0.3664	0.1413	0.032*	
C2	0.5257 (3)	0.6069 (2)	0.4118 (3)	0.0253 (6)	
C15	1.0601 (4)	0.4429 (3)	−0.2370 (3)	0.0336 (7)	
H15	1.0942	0.4472	−0.3486	0.04*	
C17	0.9776 (4)	0.5275 (2)	0.0182 (4)	0.0305 (6)	
H17	0.9552	0.5892	0.0774	0.037*	
C3	0.3739 (3)	0.5360 (3)	0.3417 (4)	0.0313 (6)	
H3A	0.3361	0.4894	0.4307	0.038*	
H3B	0.2736	0.5803	0.3088	0.038*	
C6	0.5155 (4)	0.6160 (2)	0.6049 (3)	0.0297 (6)	
H6A	0.5983	0.67	0.6438	0.036*	
H6B	0.3972	0.6394	0.632	0.036*	
C4	0.4252 (3)	0.4704 (3)	0.1921 (4)	0.0315 (6)	
H4A	0.3276	0.4244	0.1581	0.038*	
H4B	0.4511	0.5161	0.0979	0.038*	
C16	1.0257 (4)	0.5336 (3)	−0.1465 (4)	0.0386 (7)	
H16	1.0354	0.5992	−0.198	0.046*	
C9	0.5120 (5)	0.7282 (3)	0.1467 (4)	0.0435 (8)	
H9A	0.5013	0.8009	0.1143	0.065*	
H9B	0.4147	0.6888	0.0975	0.065*	
H9C	0.6218	0.7003	0.1075	0.065*	
C7	0.5558 (5)	0.5153 (3)	0.7018 (4)	0.0397 (7)	
H7A	0.5463	0.5282	0.8205	0.06*	
H7B	0.6741	0.4923	0.6791	0.06*	
H7C	0.4726	0.4615	0.6671	0.06*	
H1O	1.120 (5)	0.249 (3)	0.448 (5)	0.041 (10)*	

### Atomic displacement parameters (Å^2^)

**Table:**

	*U*^11^	*U*^22^	*U*^33^	*U*^12^	*U*^13^	*U*^23^
O1	0.0195 (9)	0.0215 (9)	0.0361 (9)	−0.0021 (8)	0.0025 (7)	−0.0075 (8)
C10	0.0156 (11)	0.0178 (12)	0.0249 (12)	−0.0015 (11)	0.0012 (9)	−0.0026 (10)
O2	0.0233 (10)	0.0281 (11)	0.0410 (11)	0.0065 (8)	0.0035 (8)	0.0138 (9)
C12	0.0132 (10)	0.0231 (13)	0.0264 (12)	0.0017 (11)	−0.0011 (9)	−0.0001 (11)
C1	0.0220 (12)	0.0193 (12)	0.0191 (10)	−0.0027 (11)	0.0022 (9)	0.0019 (10)
N1	0.0150 (10)	0.0189 (10)	0.0241 (10)	−0.0002 (9)	0.0010 (8)	−0.0023 (8)
C11	0.0199 (12)	0.0237 (13)	0.0261 (12)	0.0015 (11)	0.0057 (9)	0.0023 (10)
C13	0.0348 (14)	0.0236 (14)	0.0296 (13)	0.0064 (13)	0.0032 (10)	0.0003 (12)
C8	0.0245 (14)	0.0235 (14)	0.0494 (18)	0.0044 (12)	0.0018 (12)	0.0016 (13)
C14	0.0383 (16)	0.0343 (16)	0.0280 (14)	0.0090 (14)	0.0039 (12)	−0.0061 (13)
C5	0.0186 (12)	0.0239 (13)	0.0366 (14)	−0.0026 (11)	−0.0005 (10)	−0.0079 (11)
C2	0.0196 (12)	0.0232 (13)	0.0334 (13)	0.0009 (11)	0.0061 (10)	−0.0010 (11)
C15	0.0328 (14)	0.0463 (18)	0.0221 (12)	−0.0049 (14)	0.0062 (10)	0.0002 (12)
C17	0.0358 (15)	0.0225 (14)	0.0335 (14)	−0.0033 (13)	0.0060 (11)	0.0001 (12)
C3	0.0152 (11)	0.0356 (16)	0.0431 (15)	−0.0001 (12)	0.0035 (10)	−0.0032 (13)
C6	0.0262 (13)	0.0282 (14)	0.0353 (14)	−0.0004 (13)	0.0106 (10)	−0.0054 (12)
C4	0.0174 (12)	0.0350 (15)	0.0420 (15)	−0.0029 (12)	−0.0017 (11)	−0.0084 (13)
C16	0.0516 (19)	0.0298 (16)	0.0347 (15)	−0.0089 (15)	0.0054 (13)	0.0070 (13)
C9	0.0451 (19)	0.0331 (17)	0.0517 (19)	0.0011 (15)	−0.0065 (14)	0.0105 (14)
C7	0.0446 (17)	0.0420 (18)	0.0330 (15)	0.0031 (16)	0.0104 (13)	0.0049 (14)

### Geometric parameters (Å, º)

**Table:**

O1—C1	1.239 (3)	C5—H5A	0.97
C10—N1	1.471 (3)	C5—H5B	0.97
C10—C12	1.513 (3)	C2—C6	1.540 (3)
C10—C11	1.534 (3)	C2—C3	1.544 (4)
C10—H10	0.98	C15—C16	1.384 (5)
O2—C11	1.419 (3)	C15—H15	0.93
O2—H1O	0.80 (4)	C17—C16	1.368 (4)
C12—C13	1.387 (4)	C17—H17	0.93
C12—C17	1.390 (4)	C3—C4	1.508 (4)
C1—N1	1.343 (3)	C3—H3A	0.97
C1—C2	1.534 (3)	C3—H3B	0.97
N1—C5	1.471 (3)	C6—C7	1.515 (4)
C11—H11A	0.97	C6—H6A	0.97
C11—H11B	0.97	C6—H6B	0.97
C13—C14	1.383 (4)	C4—H4A	0.97
C13—H13	0.93	C4—H4B	0.97
C8—C9	1.508 (5)	C16—H16	0.93
C8—C2	1.550 (4)	C9—H9A	0.96
C8—H8A	0.97	C9—H9B	0.96
C8—H8B	0.97	C9—H9C	0.96
C14—C15	1.371 (5)	C7—H7A	0.96
C14—H14	0.93	C7—H7B	0.96
C5—C4	1.500 (4)	C7—H7C	0.96
			
N1—C10—C12	110.53 (18)	C1—C2—C8	107.6 (2)
N1—C10—C11	109.28 (19)	C6—C2—C8	108.0 (2)
C12—C10—C11	115.4 (2)	C3—C2—C8	110.3 (2)
N1—C10—H10	107.1	C14—C15—C16	119.2 (2)
C12—C10—H10	107.1	C14—C15—H15	120.4
C11—C10—H10	107.1	C16—C15—H15	120.4
C11—O2—H1O	105 (3)	C16—C17—C12	121.1 (3)
C13—C12—C17	118.0 (2)	C16—C17—H17	119.4
C13—C12—C10	123.7 (2)	C12—C17—H17	119.4
C17—C12—C10	118.3 (2)	C4—C3—C2	113.4 (2)
O1—C1—N1	120.7 (2)	C4—C3—H3A	108.9
O1—C1—C2	119.3 (2)	C2—C3—H3A	108.9
N1—C1—C2	120.0 (2)	C4—C3—H3B	108.9
C1—N1—C10	119.4 (2)	C2—C3—H3B	108.9
C1—N1—C5	124.0 (2)	H3A—C3—H3B	107.7
C10—N1—C5	116.61 (19)	C7—C6—C2	115.2 (2)
O2—C11—C10	107.05 (19)	C7—C6—H6A	108.5
O2—C11—H11A	110.3	C2—C6—H6A	108.5
C10—C11—H11A	110.3	C7—C6—H6B	108.5
O2—C11—H11B	110.3	C2—C6—H6B	108.5
C10—C11—H11B	110.3	H6A—C6—H6B	107.5
H11A—C11—H11B	108.6	C5—C4—C3	109.3 (2)
C14—C13—C12	120.7 (3)	C5—C4—H4A	109.8
C14—C13—H13	119.6	C3—C4—H4A	109.8
C12—C13—H13	119.6	C5—C4—H4B	109.8
C9—C8—C2	116.7 (2)	C3—C4—H4B	109.8
C9—C8—H8A	108.1	H4A—C4—H4B	108.3
C2—C8—H8A	108.1	C17—C16—C15	120.4 (3)
C9—C8—H8B	108.1	C17—C16—H16	119.8
C2—C8—H8B	108.1	C15—C16—H16	119.8
H8A—C8—H8B	107.3	C8—C9—H9A	109.5
C15—C14—C13	120.5 (3)	C8—C9—H9B	109.5
C15—C14—H14	119.7	H9A—C9—H9B	109.5
C13—C14—H14	119.7	C8—C9—H9C	109.5
N1—C5—C4	111.6 (2)	H9A—C9—H9C	109.5
N1—C5—H5A	109.3	H9B—C9—H9C	109.5
C4—C5—H5A	109.3	C6—C7—H7A	109.5
N1—C5—H5B	109.3	C6—C7—H7B	109.5
C4—C5—H5B	109.3	H7A—C7—H7B	109.5
H5A—C5—H5B	108	C6—C7—H7C	109.5
C1—C2—C6	106.26 (19)	H7A—C7—H7C	109.5
C1—C2—C3	114.4 (2)	H7B—C7—H7C	109.5
C6—C2—C3	110.0 (2)		
			
N1—C10—C12—C13	−116.5 (3)	O1—C1—C2—C3	176.7 (2)
C11—C10—C12—C13	8.1 (3)	N1—C1—C2—C3	−4.9 (3)
N1—C10—C12—C17	65.3 (3)	O1—C1—C2—C8	53.7 (3)
C11—C10—C12—C17	−170.1 (2)	N1—C1—C2—C8	−127.9 (2)
O1—C1—N1—C10	3.5 (3)	C9—C8—C2—C1	67.1 (3)
C2—C1—N1—C10	−174.9 (2)	C9—C8—C2—C6	−178.6 (2)
O1—C1—N1—C5	−177.1 (2)	C9—C8—C2—C3	−58.4 (3)
C2—C1—N1—C5	4.5 (3)	C13—C14—C15—C16	−1.3 (4)
C12—C10—N1—C1	−110.0 (2)	C13—C12—C17—C16	−0.2 (4)
C11—C10—N1—C1	121.9 (2)	C10—C12—C17—C16	178.2 (3)
C12—C10—N1—C5	70.6 (3)	C1—C2—C3—C4	−25.8 (4)
C11—C10—N1—C5	−57.5 (3)	C6—C2—C3—C4	−145.3 (3)
N1—C10—C11—O2	−167.33 (19)	C8—C2—C3—C4	95.7 (3)
C12—C10—C11—O2	67.4 (3)	C1—C2—C6—C7	−56.3 (3)
C17—C12—C13—C14	−0.3 (4)	C3—C2—C6—C7	68.0 (3)
C10—C12—C13—C14	−178.5 (2)	C8—C2—C6—C7	−171.5 (2)
C12—C13—C14—C15	1.0 (5)	N1—C5—C4—C3	−55.8 (3)
C1—N1—C5—C4	26.7 (3)	C2—C3—C4—C5	55.9 (3)
C10—N1—C5—C4	−153.9 (2)	C12—C17—C16—C15	−0.1 (5)
O1—C1—C2—C6	−61.8 (3)	C14—C15—C16—C17	0.8 (4)
N1—C1—C2—C6	116.6 (2)	C1—C5—N1—C10	179.4 (3)

### Hydrogen-bond geometry (Å, º)

Cg1 is the centroid of the C12–C17 ring.

**Table:**

*D*—H···*A*	*D*—H	H···*A*	*D*···*A*	*D*—H···*A*
O2—H1*O*···O1^i^	0.80 (4)	1.95 (4)	2.745 (3)	170 (4)
C8—H8*B*···O1	0.97	2.55	2.874 (3)	100
C10—H10···O1	0.98	2.23	2.707 (3)	108
C4—H4*A*···*Cg*1^ii^	0.96	2.96	3.723 (3)	137

Symmetry codes: (i) −*x*+2, *y*−1/2, −*z*+1; (ii) *x*+1, *y*, *z*.
